# Editorial: Molecular mechanisms of lung endothelial permeability, vol II

**DOI:** 10.3389/fphys.2024.1508729

**Published:** 2024-10-22

**Authors:** Alexander Verin, Rahul S. Patil, Narasimham Parinandi, Evgenia Gerasimovskaya

**Affiliations:** ^1^ Vascular Biology Center, Medical College of Georgia, Augusta University, Augusta, GA, United States; ^2^ Division of Pulmonary, Critical Care, and Sleep Medicine, Department of Internal Medicine, The Ohio State University Wexner Medical Center, Columbus, OH, United States; ^3^ Department of Pediatrics, University of Colorado Denver, Aurora, CO, United States

**Keywords:** endothelial cells, acute lung injury, acute respiratory distress syndrome, nuclear factor of activated T cell cytoplasmic member 3, purinergic signaling, heat shock proteins

Endothelial cells (ECs), due to their strategic location, play a central role in the pathophysiology of cardiovascular and lung diseases. In particular, disruption of vascular barrier integrity and excessive inflammation are key features of endothelial dysfunction underlying acute lung injury (ALI) and acute respiratory distress syndrome (ARDS). This Research Topic presents a unique blend of original research article, opinion, regular review, and systematic review that highlight advances in the molecular and cellular mechanisms regulating the microvascular endothelial barrier, as well as offering a systematic bibliometric analysis of the research landscape in ALI and ARDS.

The interplay of extracellular vehicles (EVs), endothelial cell responses, and signaling pathways presents a complex picture of the mechanisms underlying acute lung injury (ALI) and acute respiratory distress syndrome (ARDS). These severe respiratory conditions, complicated by intricate pathogenesis, highlight the role of EVs in mediating intercellular communication and represent potential for novel therapeutic approaches. In their study for the current Research Topic, Karpurapu et al. reported that EVs are crucial in transporting biologically active macromolecules between cells, particularly in the lungs. In mouse models, the activation of nuclear factor of activated T cell cytoplasmic member 3 (NFATc3) in pulmonary macrophages in response to lipopolysaccharide (LPS) has been observed. The inhibition of this activation by a novel cell-permeable calcineurin peptide inhibitor, CNI103, effectively mitigates the symptoms of ALI. EVs from the bronchoalveolar lavage fluid of LPS-treated mice contain elevated levels of arachidonic acid metabolites, directly implicating pro-inflammatory lipid mediators in lung inflammation and injury. This discovery points to a regulatory mechanism where the calcineurin-NFATc3 pathway influences the lipid content of EVs, impacting the pathogenesis of ALI.

The purinergic signaling system is emerging as a critical regulatory circuit, essential for maintaining homeostatic balance and modulating pathological vascular responses in various cardiovascular and lung diseases. Despite its recognized significance, investigations into purinergic regulation specifically within the lung endothelium and vasa vasorum endothelium (VVE) have remained limited. The review by Gerasimovskaya et al. highlights the protective role of purinergic signaling in pulmonary microvascular ECs, focusing on the specific signaling mechanisms of the barrier-protective effects of extracellular ATP, ATPγS, and adenosine. In addition, it provides a comprehensive overview of ATP- and adenosine-mediated regulation of angiogenic and barrier-protective responses in pulmonary artery VVECs, emphasizing a role of PY2-mediated signaling in VVEC angiogenic expansion. The review also underscores the endothelial diversity of purinergic signaling in barrier regulation and presents novel research directions and potential therapeutic targets for managing pulmonary diseases.

Among the multifaceted regulatory networks involved in pathological responses within the microvascular endothelium, Heat Shock Proteins (HSPs) have emerged as key players. The opinion article by Barabutis highlights the therapeutic approaches targeting ALI and ARDS, which involve the use of Hsp90 inhibitors to regulate endothelial barrier integrity. These inhibitors block transcription factors that drive inflammatory responses while enhancing mechanisms responsible for cellular homeostasis, making them promising candidates for treating lung inflammatory diseases. The relationship between Hsp90 inhibitors, the unfolded protein response, and the restoration of endothelial barrier function opens new pathways for addressing severe inflammation in respiratory conditions. Their potential role in modulating responses to viral infections, such as COVID-19, further underscores their utility in clinical settings.

A Systematic Review, presented by Zhou et al. highlights the increasing academic focus on the role of ECs in on ALI/ARDS. Using comprehensive bibliometric analysis, including the Web of Science Core Collection (WoSCC) and specialized bioinformatics visualization software, the authors identified leading research institutions, prominent scientists, and cross-continental collaborative networks in the field of ALI and ARDS. Additionally, they pinpointed key trends and emerging research hotspots, such as endothelial glycocalyx, oxidative stress, and pyroptosis. These insights are critical for guiding future research efforts and may help inform the development of new treatments and intervention strategies.

Together, these areas of research provide a comprehensive view of the current understanding and innovative therapeutic strategies in the field of ALI and ARDS. By integrating findings from the roles of EVs, bibliometric analyses, signaling pathways, and new therapeutic agents, the scientific community can better approach the challenges posed by these severe respiratory conditions. Future studies should focus on these integrative mechanisms and therapeutic potentials to develop more effective interventions for patients suffering from ALI and ARDS.

